# Supplementary material for: Atomic resolution mechanism of ligand binding to a solvent inaccessible cavity in T4 lysozyme
Source: PLoS Comput Biol. 2018 May 18;14(5):e1006180. doi: 10.1371/journal.pcbi.1006180 (PMC5979041; doi:10.1371/journal.pcbi.1006180)
Supplement: S1 Supporting Information — (PDF) [file pcbi.1006180.s001.pdf]

Supporting Information for "Atomic resolution mechanism of ligand binding to a solvent inaccessible cavity in T4 lysozyme"

Jagannath Mondal[1*], Navjeet Ahalawat[1], Subhendu Pandit[1], Lewis E Kay[2,3] and Pramodh Vallurupalli[1*]

[1]Tata Institute of Fundamental Research, Hyderabad, India,

[2]Departments of Molecular Genetics, Biochemistry and Chemistry, University of Toronto, Toronto, Ontario, Canada

[3]Hospital for Sick Children Program in Molecular Medicine, Toronto, Ontario, Canada

[*]Corresponding authors

Email: jmondal@tifrh.res.in and pramodh@tifrh.res.in

Supporting Figures:

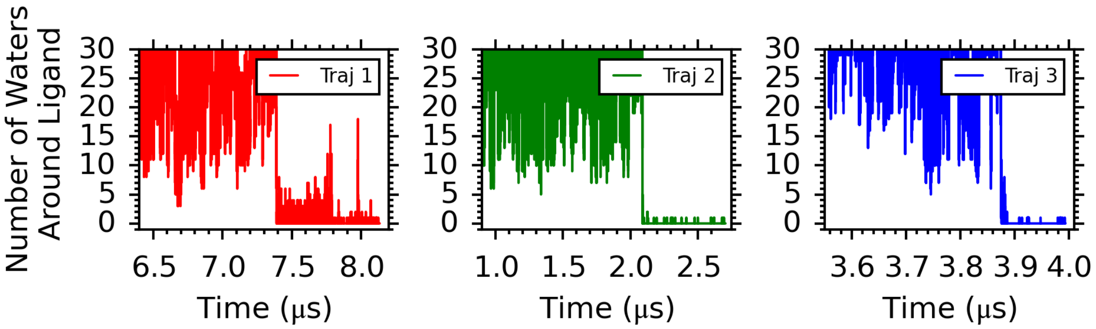

Fig. A. Binding of benzene to the cavity of T4L L99A results in desolvation in long unbiased MD simulations. Number of water molecules within a spherical radius of 0.5 nm of any carbon atom of benzene is computed as a function of time. The time profile shows a gradual decrease in the number of waters surrounding benzene as the ligand approaches the solvent-inaccessible cavity, leading to complete dewetting upon binding.

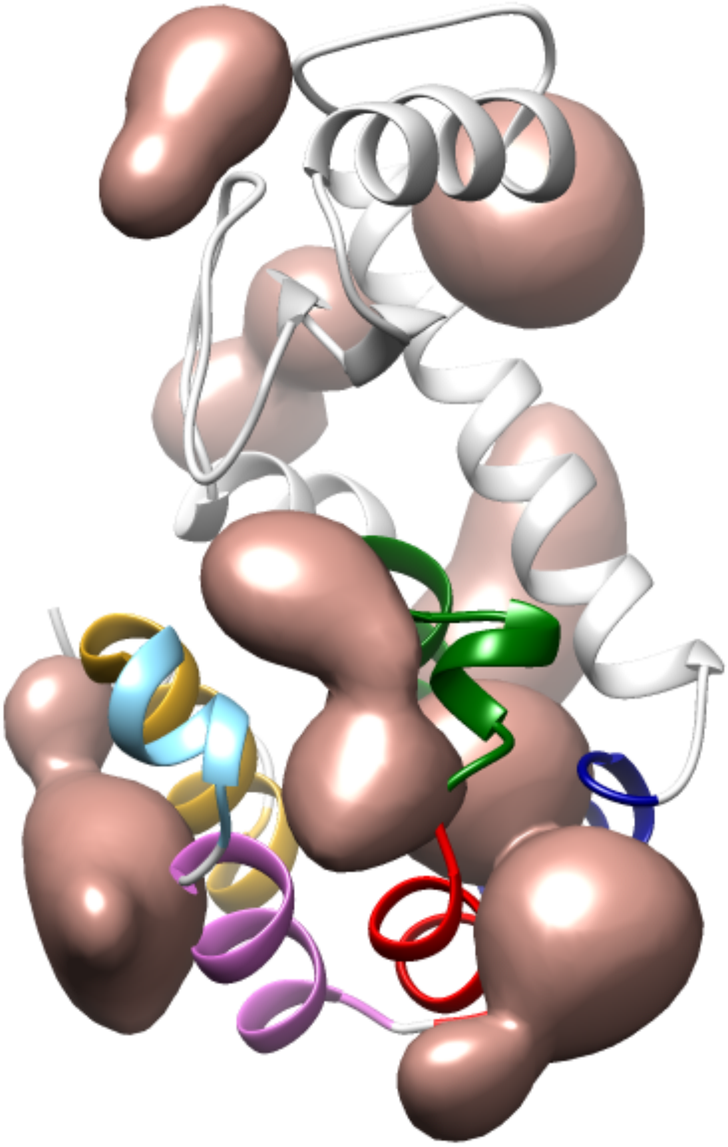

Fig. B. Spatial density of benzene around T4L L99A. A cutoff of 0.6 nm from any protein heavy atom is used to compute instances of benzene contacting the protein, from which the ligand density profile is calculated. Although the majority of the density is concentrated near the cavity and the C-terminus helices (helices 4-9), there are also locations far from the cavity where benzene can reside for significant amounts of time. Helices are colored as in the rest of the paper.

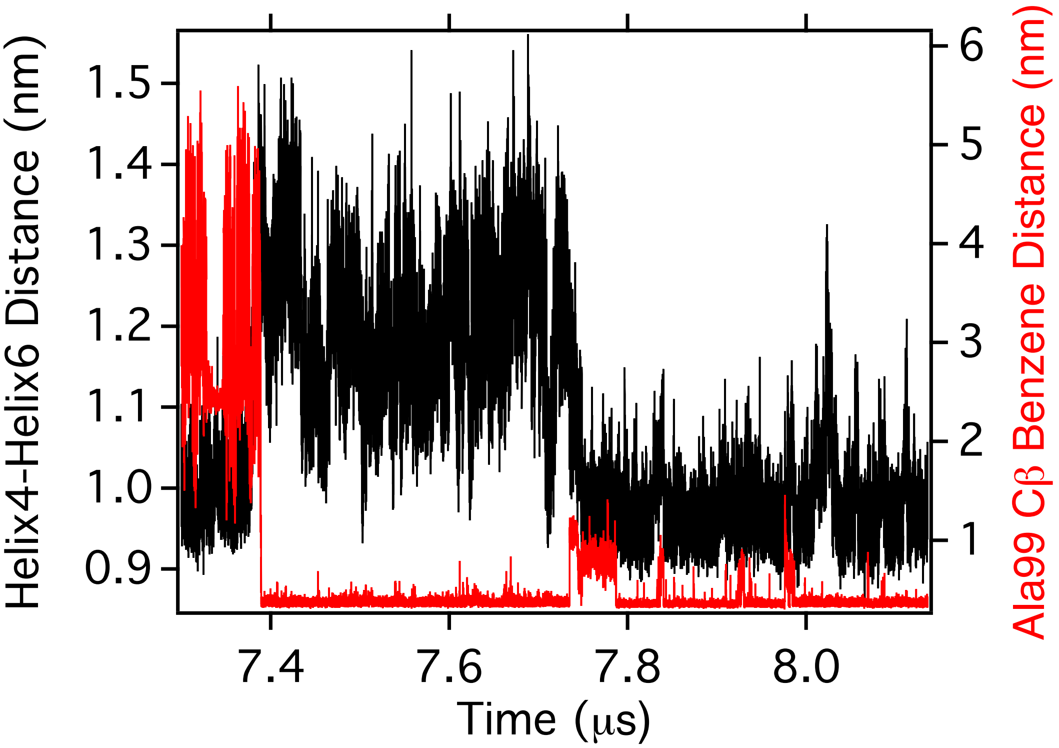

Fig. C. A longer timescale plot of Figure 3B. The time-profile of the distance between helices 4 and 6 (left-hand axis) and the progression of the cavity-ligand distance (right-hand axis) for L99A T4L are shown. The helix4-helix6 distance increases (to ~1.3 nm) prior to entry of the ligand into the cavity, reverting back to its equilibrium value (~1.0 nm) after the ligand binds to the pocket.

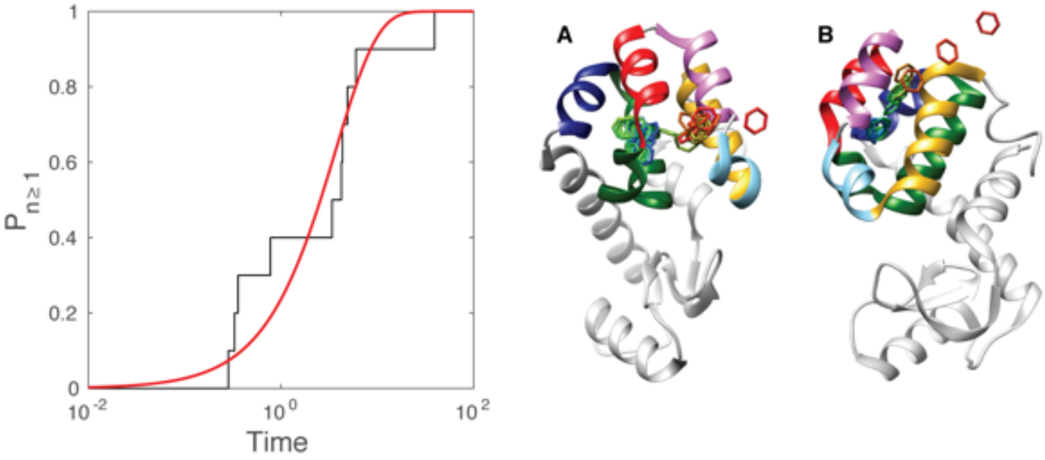

Fig. D. Unbinding process simulated via infrequent metadynamics techniques (25): Left: Poisson fit analysis for the unbinding process. The black and red curves denote the empirical and theoretical (i.e. best fit) cumulative distribution functions, respectively. The p-value of 0.72 suggests a good Poisson fit, well above the statistical threshold of 0.05, implying that the metadynamics simulation was infrequent enough such that the transition state was not influenced from biasing (25,26). The unbinding rate obtained from the analysis is 369 s^{-1}, in reasonably good agreement with that measured from experiment. Right: Unbinding pathways obtained from the infrequent metadynamics simulation add to the insight obtained from the binding pathways based on unbiased MD simulations. In pathway A benzene unbinds at the junction between helices 5, 6, 7, 8, while in pathway B, ligand is extruded between helices 7 and 9. Coloring of helices is as in Figure 1. Benzene is colored in blue (bound) and finally denoted in red when it is removed from the protein.

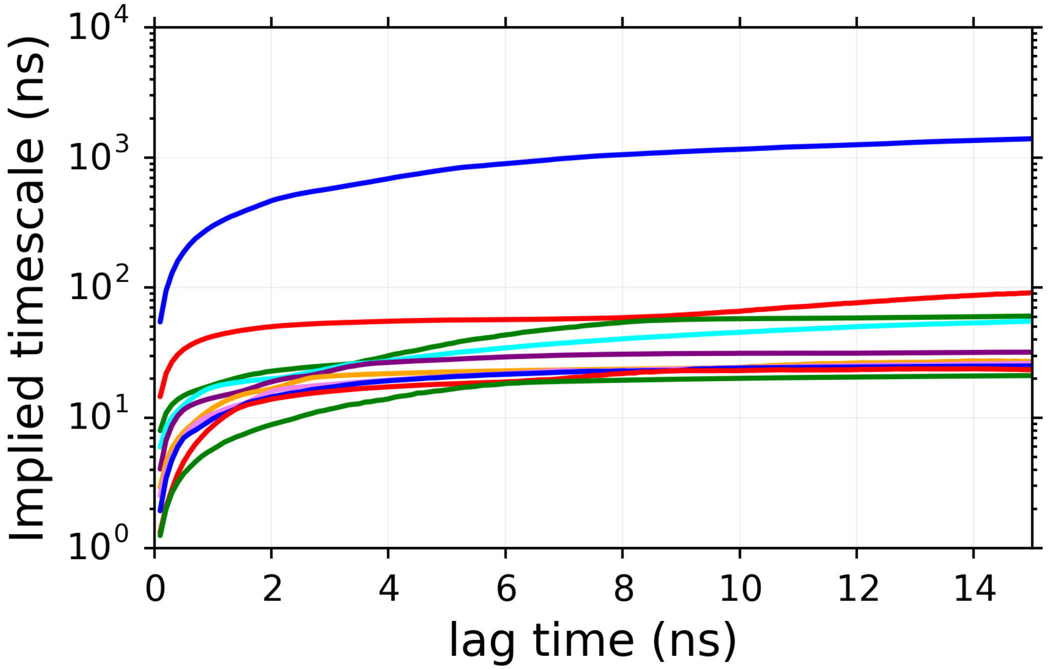

Fig. E: **Plot of Implied time scale as a function of lag-time as obtained during construction of the Markov State Model.** The value of implied time scale (only top 10 shown), calculated at every 100ps, reaches a plateau at around a lag-time of 10 ns, which has been used to build the Markov State Model described in the main text.
